# Castleman's disease in childhood: report of three cases and review of the literature

**DOI:** 10.1186/1824-7288-37-50

**Published:** 2011-10-20

**Authors:** Piero Farruggia, Antonino Trizzino, Nunzia Scibetta, Giovanni Cecchetto, Patrizia Guerrieri, Emanuele SG D'Amore, Paolo D'Angelo

**Affiliations:** 1 Unit of Pediatric Hematology and Oncology, "G. Di Cristina" Children's Hospital, A.R.N.A.S., Palermo, Italy; 2 Unit of Pathology, "Civico e Benfratelli" Hospital, A.R.N.A.S., Palermo, Italy; 3 Pediatric Surgery Department, University of Padua, Padua, Italy; 4 Unit of Oncological Radiotherapy, "Civico e Benfratelli" Hospital, A.R.N.A.S., Palermo, Italy; 5 Unit of Pathology, San Bortolo Hospital, Vicenza, Italy

**Keywords:** Castleman's disease, childhood, differential diagnosis, histological subgroups, outcome

## Abstract

Castleman's disease (CD) is a rare, localized or generalized, lymphoproliferative disorder with a frequent mediastinal location, but possible in any lymph node or extra nodal site. It usually appears in young adults whilst it rarely occurs in childhood. There are only about 100 pediatric cases published, five of them in Italy. We report 3 cases of localized Castleman's disease, investigated in our Department in a 3 years period and reviewed the literature.

## Background

Castleman's disease (CD) is a rare and not well recognized disease characterized by a massive growth of lymphoid tissue of unknown etiology that was first described in 1954, and subsequently better defined by Castleman in 1956 [1,2]. The disease usually presents in young adults and is probably slightly more frequent in women. CD is classified into two clinical subtypes: a localized and a multifocal subtype. CD may occur anywhere along the lymphatic system, although the most common location (70%) is the mediastinum. Extrathoracic sites have been reported in the neck, axilla, pelvis and retroperitoneum [3]. Surgery is the optimal therapeutic approach only in the localized form, while for unresectable or disseminated disease, partial surgical resection, steroid, chemotherapy and radiotherapy have been employed with some measurable success.

We report three cases of localized hyaline-vascular CD in pediatric age observed in our Department in the last 3 years; a complete surgical resection was not feasible in one of them and so various therapeutic attempts were performed.

## Case Reports

### Case 1

**Figure 1:**
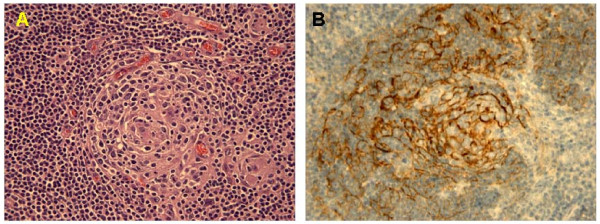
Histology showed a follicular center exihibiting prominent hyalinized vessels, surrounded by concentric layers of lymphocytes **(A)**, dendritic follicular cells in the germinal center were positive for CD21 **(B)**.

GM, female, was admitted, at the age of 3.3 years, with an asymptomatic left axillary mass showing a slow progressive growth. At presentation she was in good general conditions and the physical examination was normal. Routine blood tests were all normal too. Radiological examinations (chest X-ray, abdominal ultrasound, chest CT scan) confirmed the adenopathy and excluded other locations. After 2 months a complete surgical resection was performed, and a single lymph node of 4 cm maximum diameter was removed. Histological assessments indicated the diagnosis of hyaline-vascular CD (Figure 1A,B). There has been no recurrence 48 months after diagnosis.

### Case 2

**Figure 2:**
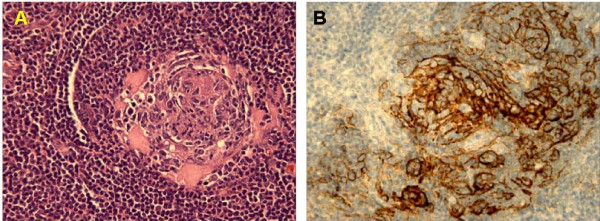
Histology showed small hyalinized germinal center within an expanded mantle zone **(A)**, dendritic follicular cells in the germinal center were CD21-positive **(B)**.

LG, male; at the age of 3.8 years an isolated palpable subcutaneous tumefaction, sized 1 cm in maximum diameter, appeared on his right blade shoulder. On admission he was in good physical condition; routine blood tests, abdominal ultrasound and chest X-ray were all normal. After a follow-up period of 3 months, during which the mass enlarged to a diameter of 2.5 cm, a complete surgical resection was performed and two subcutaneous and intramuscular nodules, having maximum diameter 2.5 and 1.2 cm respectively, were removed and examined. Histology revealed a classic picture of hyaline-vascular CD (Figure 2A,B). No recurrence was noted after a 36-month follow up.

### Case 3

**Figure 3:**
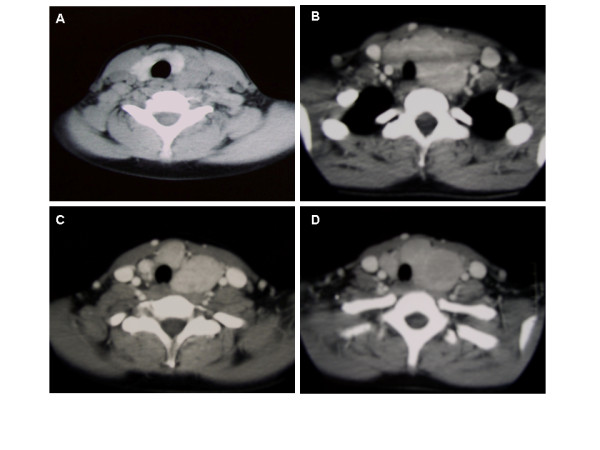
CT scan showed that the neck mass, a multiple lymph node enlargement, with an high contrast-enhancement, originated in the anterior-superior mediastinum and cause a serious right dislocation of the trachea **(A-D)**.

**Figure 4:**
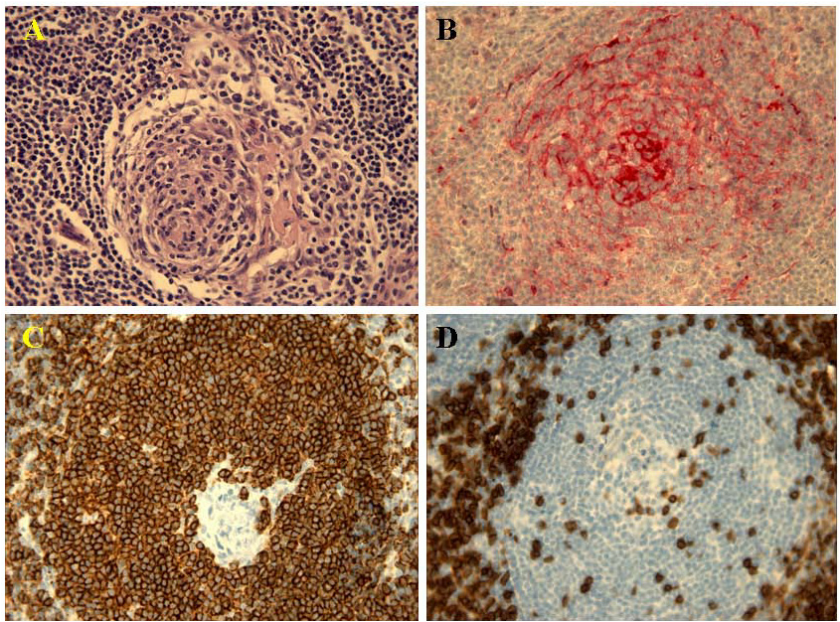
Histology showed small germinal center depleted of follicular center B cells, and penetration by hyalinized venules (A); follicular dendritic cell were evidenced by their strong immunoreactivity for CD 35 (B); immunoreactivity for CD20 (C) and CD3 (D) was strongly positive.

DA, male; at the age of 13 years a mass in the anterior part of the neck was noted. He was admitted to an endocrinology department because a thyroid tumor had been suspected. A chest computed tomography (CT) scan showed that the neck mass, with high contrast-enhancement, originated in the anterior-superior mediastinum and caused a serious right dislocation of the trachea (Figure 3A-D). A fine needle biopsy of this mass was promptly performed, but it was not diagnostic, with the detection of small normal lymphocytes only: consequently the patient was referred to our Unit. The boy was in good general condition, without any symptom as fever, night sweats or weakness, and the physical examination, excluding the palpable mass of the anterior part of the neck, was normal. All the blood tests, particularly serum levels of LDH, ERS, CRP, ferritin, copper, immunoglobulins and IL-6 were within normal range: IL-6 was tested because of the atypical presentation of a suspected lymphoma (no constitutional symptoms, no superficial lymphadenopathy and a soft consistency of the tumor). Abdominal ultrasound and^ 99 m^Tc-MDP total body bone scan were both normal. After two weeks, suspecting a mediastinal Hodgkin Lymphoma, an open biopsy of the mass was performed; during the surgical procedure massive bleeding occurred and the hemoglobin level dropped from 12.3 to 8.7 gr/dl. No other serious events occurred. The histology showed the typical aspect of a hyaline-vascular CD (Figure 4A-D). A second neck-chest CT scan and echocardiography confirmed the tumor was not resectable, so, in consideration of a formal request of treatment by the parents, a prednisone treatment (25 mg × 3/day p.o. for two months) was started: a chest CT evaluation revealed it was completely ineffective. The same poor result was obtained by chemotherapy with vinblastine i.v. at 10 mg/week for 8 doses.

After prolonged and troublesome discussion with surgeons, an attempt of surgical excision was planned through a left trap door incision. This approach consisted of a left-anterior cervical access, median sternotomy and left-anterior thoracotomy that could provide adequate exposure of the neurovascular structures [4]. The mass had a vascular aspect and encased the left internal jugular vein and the carotid artery down into the mediastinum. Repeated attempts to remove the lesion caused massive and life-threatening bleeding; therefore the procedure was prematurely interrupted and an abundant transfusion of red blood cells was performed.

Finally, after troubling discussion with the parents, the boy underwent a 45 Gy course of 3D conformal radiation therapy on the upper mediastinal mass, with standard fractionation of 1.8 Gy per day. The treatment was delivered through two antero-posterior, postero-anterior asymmetrically weighted beam pairs of different energies in order to encompass the mass into the 95% isodose. Particular care was taken to avoid undue radiation delivery to the nearby organs: right and left lungs received an average dose of 8 and 6.4 Gy respectively, while the spinal cord maintained a maximum dose less than 45 Gy. A CT scan performed at two months from the end of radiotherapy showed a reduction of the mass by about 60% and a significant improvement of tracheal dislocation: both were more evident in a second CT scan performed 3 months later. The FDG-PET scan that initially revealed a mildly significant uptake of the radiopharmaceutical of the residual disease at three months from the end of radiotherapy, became totally negative after a further 3 months, confirming the effectiveness of our therapy choice. A further reduction of the residual mass sizes and negative total body scan was obtained six months later with neck-chest CT scan and FDG-PET scan respectively. Now the boy is well, on a watchful follow-up, without any side effect of radiotherapy, 12 months after discontinuation of treatment.

## Pathology Findings

Histologically all of our cases, diagnosed as hyaline-vascular type of Castleman's disease, showed a mass of lymphoid tissue with scattered abnormal lymphoid follicles, which ranged in size from small to large. The germinal centers were depleted and composed predominantly of follicular dendritic cells and endothelial cells of hyalinized capillaries. Their appearance corresponds to that of "hyaline vascular nodules". Another peculiar finding was the presence of more than one small germinal center within a single follicle. There was a concentric layering of lymphocytes at the periphery of the follicles with an onion-skin appearance.

The interfollicular areas showed vascular proliferation with numerous hyperplastic vessels of the postcapillary venule type with hyaline material between vessels and absence of sinuses. A small number of lymphocytes, plasmacells, eosinophils and immunoblasts were present.

Immunohistochemically, positive CD21 and CD35 were seen in the large cells with vesicular nuclei (follicular dendritic cells) in the center of follicles, strong positive FVIII-related antigen was seen in the endothelium of the interfollicular vessels, but only weak and focal reaction was found in the hyaline vessels located in the center of the follicles.

The immunoglobulin production by plasmacells was polytypic and in the interfollicular areas large numbers of suppressor T cells were found.

In summary the diagnostic features were small hyaline germinal centers within an expanded mantle zone, as well as a highly vascularized interfollicular network.

## Discussion

CD is a disease of unknown etiology, first described by Castleman in 1956 [2] and currently classified in two subgroups: localized (only one group of lymph nodes involved) and disseminated (two or more lymph nodal groups involved). Localized disease seems to be much more frequent, above all in the case of thoracic presentation [4,5]. There are three major histological subtypes: hyaline-vascular CD (HV-CD), plasma cell CD (PC-CD) and a plasmablastic variant associated with HHV8 and HIV. The first is much more frequent (91-96%) [5,6]. The majority (57-91%) of localized disease is hyaline-vascular [7,8]. Most cases of disseminated CD are PC-CD variants or plasmablastic variants in HIV+ patients [8,9]. The prognosis of the disseminated PC-CD is worse whereas disseminated variants with HV-CD histology or with a mixed appearance between HV-CD and PC-CD seem to have a better course [3,8-11].

Prevalence is estimated to be less than 1/100,000 [12]. There are some series where female sex is predominant [4,13,14], but in many others there is no sex predilection [6-8]. The peak of incidence is in the third and fourth decade of life for localized CD and in the fourth and fifth decade of life for disseminated CD [7,8,13].

It is postulated that the disease represents a reaction to chronic viral antigenic stimulation with the bulk of evidence pointing toward an interleukin-6 role, above all in the form of the disease associated with systemic manifestations [15-17]. The large majority of cases don't show lymphocyte monoclonality [18]. In many cases the HHV-8 has been postulated as the virus capable of causing the disease, above all in multicentric cases [19,20].

Finally, there are many reports of association of the multicentric type with HIV infection with a global incidence of 4.3/10,000 patient-years [21,22].

The majority of HV-CD at presentation is asymptomatic but, in some cases, fever, night sweat or weight loss can be present; in contrast, if the localized disease has a plasma cell histology, constitutional symptoms are very frequent, as in the disseminated form [7,8]. The site of presentation can be abdominal, mediastinal or peripheral and the frequency of involvement reported in the literature is variable [6,8,23]. The CD located in the thorax can be found in mediastinum (anterior above all, but middle and posterior too) and, rarely, in the chest wall, pleura, pericardium, intercostal space, and lung [5,24,25]. Usually the mediastinal masses are wide and most of the thoracic cases are asymptomatic at presentation; sometimes there are symptoms connected to local pressure from the mass (cough, chest pain, dyspnoea etc.). Invasion and adherence to vessels and bronchi are common patterns and often the mass proves difficult to complete resection, as in our case [5]. When the location is abdominal the presentation sometimes includes abdominal pain [26].

The plasma cells and/or the multicentric CD present with a variety of constitutional symptoms: peripheral lymphadenopathy, hepatosplenomegaly, weight loss, anemia, asthenia, night sweats, fever, skin rash, lung disorder and kidney dysfunction [8,25,27].

Less common presentations are polyneuropathy, pleural effusion, ascites etc. [23,28]. Typically there are also laboratory abnormalities: elevated serum concentration of gamma globulin and acute phase proteins, thrombocytosis, elevated ESR, low serum albumin level, proteinuria, and, sometimes, elevated IL-6, especially in the plasma cell systemic disease [8].

Enhancement on CT images after intravenous administration is universally reported but it seems that the enhancement is to a lesser degree in the plasma cell variant. At MRI the T1 weighted images are isointense or slightly hyperintense relative to the skeletal muscle; the hyperintensity is much more evident on T2 weighted images [5].

About the therapy issue, it is difficult to draw definitive conclusions for both local and disseminated CD, but some broad indications can be found.

Since the recurrence of localized CD is very unusual when the resection is complete, surgery is the preferred treatment in these cases [6-8,29-31]. When a complete resection is impossible, in the case of unicentric hyaline-vascular disease, partial resection and/or, radiation therapy with doses ranging from 27 to 45 Gy are a possibility [7,32,33]. It has been reported that with these treatments some patients can remain asymptomatic "long term" and that, even in the case of mediastinal disease, simple partial resection without any extra treatment has not been followed by recurrence [7]. Surprisingly there are very few cases where steroid therapy has been attempted, but this could be explained by the fact that complete resection is easily performed in all non-mediastinal sites and that radiotherapy is traditionally preferred in the case of mediastinal lymphoma [33]. It is important to note, however, that there are some case reports where the disease, without any treatment, remains asymptomatic or shows a gradual spontaneous improvement and even complete remission [7,8]. The location of the lymphadenopathy has no influence on the outcome [8].

Even though cases of spontaneous remission have been described [11], the course of disseminated CD, especially in the plasma cell form, is often reported poor due to complications like infections, severe autoimmune anemia, sarcoidosis, amyloidosis, POEMS syndrome (polyneuropathy, organomegaly, endocrinopathy, monoclonal antibody, skin changes), and evolution into malignant neoplasm, principally lymphoma, follicular dendritic cell sarcoma or, especially in HIV positive patients, Kaposi sarcoma [8,15,18,34,35].

In some more recent series, however, the prognosis seems to have been better [7]. The treatment of the disseminated form is not well documented but chemotherapy has been successful in many cases [3,7,8]. Regimens against Hodgkin Lymphoma are the most commonly used, even in childhood [36]. The role of radiotherapy is more uncertain than in the localized form, since there are cases where it proved to be useful and others where there was no response [11,37]. In some cases steroids alone can be sufficient for remission even though the treatment must often be prolonged; it is possible that the response to steroids can have a prognostic value [8,10,38]. Finally there are some sporadic cases where experimental therapies like autologous hematopoietic stem-cell transplantation, rituximab, valganciclovir, tocilizumab (an anti-IL-6 receptor antibody), interferon-alpha, anakinra - an IL-1RA agonist - and, more recently, siltuximab, a new anti-IL-6 chimeric monoclonal antibody, have been used with some success [21,39-44]. Since the treatment of our first two patients was an easy uncomplicated complete surgical resection, we should add some comments about therapy strategies in our third patient, that could not be surgically resected completely. When this option is impossible the most important aspects to consider are the volume and location of the mass: probably in sites not "at risk" careful observation is the best option and radiotherapy can be taken into account in other cases.

Our experience also emphasizes the potential hazards of surgical resection depending on the location and vascular characteristics of the Castleman's disease: the second surgical attempt was prematurely stopped due to serious life-threatening bleeding. Probably, a preoperative embolization could be a safety precaution before of a surgical approach on a HV-CD variant [31]. Between the two surgical procedures we first attempted a trial with steroid (prednisone 25 mg × 3/day) for 2 months, that totally failed; subsequently we performed a trial with administration of vinblastine weekly for 8 doses, without any response. After the ineffective surgical procedure we were extremely troubled in the treatment decision making and, finally, opted for local radiotherapy for the following considerations: a) some authors reported the effectiveness of lymphoma-like multiagent chemotherapy, such as CVAD or CHOP schedule, but only in multicentric CD, that represents a more aggressive clinical entity [7]; b) our patient suffered from a localized CD, and radiotherapy, as reported by some authors, can also achieve a good clinical response [45]; c) our patient was a big pubertal male (weight 68 Kg and height 171 cm), in which consequences of breast exposure to radiation or growth abnormalities are fairly improbable. On the contrary, the risk of cardiac dysfunction, growth abnormalities and secondary malignancies may limit the application of radiotherapy in other pediatric patients (pre-pubertal and females).

Thus we planned local radiotherapy, choosing a total dose of 45 Gy, that had good results, as previously reported in the literature [45]. CT scan evaluation showed a marked reduction of the mass and a significant reduction of tracheal dislocation, and there was an absolute disappearance of the radiopharmaceutical uptake in the last FDG-PET scans, performed 6 and 12 months after the end of radiation treatment.

## List of abbreviations

CD: Castleman's Disease; CT: computed tomography; LDH: lactato-dehiidrogenase; ERS: eritrosedimentation rate; CRP: c-reactive protein; IL-6: interleukin 6;^ 99 m^Tc-MDP: technetium-methil-diphosphonate; FDG-PET: phospho-di-glicerato-positron emission tomography; CD21: cluster differentiation 21; CD35: cluster differentiation 35; HHV8: human herpes virus 8; HIV: human immunodeficiency virus; MRI: magnetic resonance imaging; POEMS: polyneuropathy, organomegaly, endocrinopathy, monoclonal antibody, skin changes syndrome

## Competing interests

The authors declare that they have no competing interests.

## Authors' contributions

PF has made substantial contributions to conception and design of the manuscript, was involved in clinical management of the patient, drafted the manuscript and gave final approval of its last version. AT has made substantial contributions to conception and design of the manuscript, was involved in clinical management and helped to draft the manuscript. NS and ESGD carried out histology study, suppling all the tissue images and participated in drafting of part of the manuscript. GC was involved in surgical management of the patient, and participated in drafting of part of the manuscript. PG was involved in clinical management of the patient in the phase of radiation therapy and participated in drafting of part of the manuscript. PD has made substantial contributions to conception and design of the manuscript, was involved in clinical management of the patient, helped to draft the manuscript and gave final approval of its last version. All authors read and approved the final manuscript.

## Authors' details

^1^PF, AT and PD are physicians of Pediatric Hematology and Oncology Unit, at "G. Di Cristina" Children's Hospital, Palermo. They are AIEOP (Italian Association of Pediatric Hematology and Oncology) members. PF has a peculiar experience in the field of pediatric lymphoma and non oncologic hematology, AT has particularly developed his activity in the field of primary immunodeficiency and histiocytic disorders, while PD is especially involved in the management of children and adolescents with solid tumors.

^2^NS, from Unit of Pathology, "Civico e Benfratelli" Hospital, A.R.N.A.S., Palermo, Italy.

^3^GC, from Pediatric Surgery Department, University of Padua, is chairman of GICOP (Italian Group of Pediatric Oncologic Surgery), SIOP member and co-chairman in various international pediatric oncology trials.

^4^PG, from Unit of Oncological Radiotherapy, "Civico e Benfratelli" Hospital, A.R.N.A.S., Palermo, Italy.

^5^ESGD, from Unit of Pathology, San Bortolo Hospital, Vicenza, Italy. ESGD is one of the AIEOP referring pathologist in the field of Hodgkin and non-Hodgkin Lymphoma.

## Consent

Written informed consent was obtained from the patients' parents for publication of these case reports and any accompanying images. A copy of the written consent is available for review by the Editor-in-Chief of this journal.
